# Evaluating frontoparietal network topography for diagnostic markers of Alzheimer’s disease

**DOI:** 10.1038/s41598-024-64699-w

**Published:** 2024-06-19

**Authors:** Bayard Rogers

**Affiliations:** Department of Psychology, University of Glasgow, School of Psychology and Neuroscience, Glasgow, Scotland, UK

**Keywords:** Alzheimer’s disease, ERP, Frontoparietal, Memory, N4, P6, Topography, Diseases, Neurological disorders, Dementia, Alzheimer's disease

## Abstract

Numerous prospective biomarkers are being studied for their ability to diagnose various stages of Alzheimer’s disease (AD). High-density electroencephalogram (EEG) methods show promise as an accurate, economical, non-invasive approach to measuring the electrical potentials of brains associated with AD. Event-related potentials (ERPs) may serve as clinically useful biomarkers of AD. Through analysis of secondary data, the present study examined the performance and distribution of N4/P6 ERPs across the frontoparietal network (FPN) using EEG topographic mapping. ERP measures and memory as a function of reaction time (RT) were compared between a group of (*n* = 63) mild untreated AD patients and a control group of (*n* = 73) healthy age-matched adults. Based on the literature presented, it was expected that healthy controls would outperform patients in peak amplitude and mean component latency across three parameters of memory when measured at optimal N4 (frontal) and P6 (parietal) locations. It was also predicted that the control group would exhibit neural cohesion through FPN integration during cross-modal tasks, thus demonstrating healthy cognitive functioning consistent with older healthy adults. By targeting select frontal and parietal EEG reference channels based on N4/P6 component time windows and positivity, our findings demonstrated statistically significant group variations between controls and patients in N4/P6 peak amplitudes and latencies during cross-modal testing. Our results also support that the N4 ERP might be stronger than its P6 counterpart as a possible candidate biomarker. We conclude through topographic mapping that FPN integration occurs in healthy controls but is absent in AD patients during cross-modal memory tasks.

## Introduction

Alzheimer’s disease (AD), one of the most common forms of dementia, is a complex progressive neurodegenerative condition believed at least in part to be caused by an abnormal build-up of proteins in and around brain cells^1–3^. As these cells become affected, brain tissue atrophies, resulting in a decrease in neurotransmission and synaptic activity between different cortical regions^4,5^. There are many hypotheses about the causes of AD, and due to the complexity of the human brain, the detailed pathogenesis of this disease has not been determined. To better understand the pathophysiological processes of AD, electroencephalograms (EEGs) offer researchers a cost-effective, non-invasive tool for the detection and comparison of normal and anomalous brain activity and are becoming increasingly popular for identifying and quantifying changes in the human brain with respect to memory decline and neurodegenerative disorders^6–8^. There is ample research available on the existence of neural loops and event-related potentials in large-scale brain networks that support memory ^9–11^. Event-related potentials (ERPs) are tiny measurable voltages generated in and throughout brain structures as a direct response to a specific sensory, motor, or cognitive event^12,13^. As such, electrophysiology provides a real-time readout of neural function and network activity, allowing brain-wave measurements and brain scans of various stimuli under experimental conditions to potentially serve as functional biomarkers for AD, particularly at the group and individual levels^14^.

The study of large-scale brain networks provides researchers with a powerful paradigm for investigating neurological disorders by offering an inclusive physiologic architecture through which to explore integration. Executive control does far more than inhibiting automatic responses; it influences working and episodic memory, mediates adaptive responses, and supports a range of other executive functions by way of distributed brain networks^10^. Episodic memory is the neurocognitive system that enables human beings to recall past experiences. Specifically, self-compassion relates to personally experienced events that evaluate memory in the context of recognition, which refers to the judgement that a stimulus event has been previously experienced^15^. Thus, episodic memory exemplifies the memory of everyday events that can be summoned and explicitly detailed. Studies regarding episodic memory provide researchers valuable insight into how information is acquired, organized, and retrieved^16^. Ray, et al.^10^ examined whole-brain modular structures during tasks involving episodic memory demands. Using functional magnetic resonance imaging (fMRI), researchers have explored context-dependent brain network reorganization in healthy adults performing tasks involving cognitive control and episodic memory demands. Connections between the frontal and parietal regions were identified in regulating cognitive control demands during the encoding and retrieval of episodic memories. While their analysis yielded varying levels of network integration (and separation) during these memory-related tasks, the results support the hypothesis that engagement is largely context-driven and that the frontal and parietal regions flexibly integrate across domains to support control in episodic memory. In the context of the study, cognitive integration suggests a process in which external elements are appropriately assimilated into our cognitive loops, resulting in different degrees of connectivity demanded by encoding and retrieval during episodic memory processing^10^. Such studies provide insight into how brain networks flexibly organize and reorganize to support control during various tasks across a variety of cognitive domains. Li, et al.^17^ investigated how humans process auditory and visual stimuli through the integration of neural systems dedicated specifically to multisensory (or multimodal) information. Employing fMRI, they endeavoured to identify and localize the activation of multisensory-specific regions during categorical learning. Findings showed that brain regions once considered to play specific roles—or unimodal tasks—are often resourced outside their primary functional domains.

Nee, et al.^18^ examined the meta-analysis of the executive components of short-term working memory. The full sample of the study consisted of data from 36 experiments reporting 461 activation foci that revealed a “broad network of medial and lateral frontal and parietal regions involved in the executive processing of working memory”^18^. Researchers have long investigated age-related frontal and parietal scalp ERPs during bottom-up and top-down processing^19–22^. Taken together, these studies on memory have provided evidence that older and younger adults recruit different areas of the frontal and parietal lobes of the brain during bottom-up and top-down processing, respectively, with older adults relying on a more frontally distributed network^23^. The results of these findings suggest an organization of working memory by function, and through the process of neural association/dissociation (analogous to the work by Ray, et al.^10^), the conceptualization of executive processes being network-based becomes profound.

### The frontoparietal network (FPN)

In a seminal work by Posner and Dehaene^24^, it was maintained that attention and response in the human brain scarcely lay on one single area. Researchers have proposed that specific cognitive processes are mediated by relative electrical activity, are context dependent, and are found in specialized cortical areas spanning the frontal and parietal lobes. The frontoparietal network (FPN) is hypothesized to act as a flexible hub of cognitive control and mediation that can alter functional connectivity across neural networks based on specific objectives^10,25,26^. Most recently, Fischer, et al.^27^ sought to identify a common set of structures in the FPN involved in memory-guided attention. Their analysis yielded four significant clusters: the angular gyrus, involved in episodic memory encoding and retrieval^28–30^; the superior parietal lobe, associated with visual attention^31^; the middle frontal gyrus, related to working memory performance, action sets, and decisions^32–34^; and the mid-cingulate cortex, active in cognitive control processing^35,36^. The findings, supported by network-level interaction effects demonstrated via functional brain imaging, are consistent with the idea that retrieved memories and attentional systems are at least in part mediated by frontoparietal circuits. Such works not only demonstrate but also highlight the importance and relevance of both structure and function in complex brain networks.

In cases of dementia, early cognitive deficits in AD patients are observed in episodic memory, which encompasses the encoding, storage, and retrieval of temporally and spatially defined events and the relationships between them^37^. Auditory-visual working memory deficits, often ascribed to central executive impairment, are also recognized features of AD^38,39^. Located in the temporal lobe and involved in the consolidation of memory and learning, the hippocampus is one of the earliest affected brain regions in AD^40,41^. In the neocortex, the hippocampus functions as a rapidly adaptive structure that regulates emotions and captures episodic memories^42–44^. Furthermore, evidence suggests that the hippocampus contributes to the encoding of visual objects within auditory contexts^45,46^. An article by Eichenbaum^47^ discussed how the prefrontal cortex (PFC) and hippocampus support complementary functions in episodic memory, highlighting direct and indirect prefrontal-hippocampal pathways between the two regions as critical for mediating effective memory. These findings support earlier work by Miller and Cohen^48^ proposing an integrative theory of the PFC in cognitive roles such as memory, analogizing the PFC as a switch operator for a host of complementary brain functions. Acknowledging the broad consensus of the hippocampal system in the encoding and retrieval of episodic memories, we seek to further explore the cortical systems underlying hippocampal functions that facilitate effective memory outcomes. A system-based evaluation of cognition provides insight into how brain networks restructure in support of various tasks, as the FPN disengages and integrates as needed to support mechanisms in different domains^10^. The resulting dynamic shift between executive regions is believed to demonstrate a more concrete representation of information acquisition and analysis, suggesting the presence of cognitive flexibility in healthy adults^49^. A study by Van Buuren, et al.^50^ aimed to show how functional network interactions at rest underlie individual differences in the memory of healthy young adults. Their findings demonstrated that effective memory hinges not only on the successful connectivity of various cortical networks but also that the strength of those connections translates to better memory performance. It has become clear that successful and effective memory relies upon a number of cognitive processes spanning large-scale cortical networks. ERP components that reflect the connection process across brain regions during the course of multisensory integration mark this activity (Supplementary information).

### N4 and P6 ERPs

N4 ERP amplitudes are widely accepted as functionally sensitive in matters of semantic activation, recognition memory, predictive processing, attention, and discourse^51^. Contemplating a multitude of parameters, a significant number of AD studies have shown a lower N4 amplitude in AD-diagnosed patients than in healthy older adult controls^52–55^. As the N4 is arguably one of the more robust measures of brain activity that underscores the use of semantic memory^56^, the present study seeks to examine the degree to which it contributes to episodic memory. The results of earlier studies support N4 as being highly active in conceptual memories involving pictures and words^57^. Recognition memory is a subcategory of declarative memory and refers to the ability of the brain to recognize previously encountered stimuli as familiar^58^. From a dual-process perspective, recognition memory is conceptualized as relying on familiarity and recollection^59–61^. In the experimental setting, participants resolved recognition paradigms by making old/new recognition judgments using yes/no tests. Employing scalp-recorded ERPs during such tests enables researchers to measure activity and localization linked to these processes. While the N4 ERP has been associated with recognition memory and familiarity, it has also been linked to how predictability influences memory, specifically how the forecasting of recognition paradigms might impact the encoding of informatiomn^62–64^. Because activity across a wide network of brain areas is elicited in the N4 component time window, the highly distributed nature of this neural source makes it suitable for analysis within and between large-scale cortical networks.

The P6 is widely recognized as a language-relevant ERP thought to be elicited by hearing or reading syntactic anomalies, although modern assessments accept that the P6 reflects the general effect of processing difficulty, whether it is syntactic or semantic^65^. Shen, et al.^66^ validated the existence of semantic P6 localized in executive function areas outside of the language system, thus expanding the application of EEG to P6 as a possible reliable and sensitive biomarker for other cognitive processes, including memory. Schloerscheidt and Rugg^67^ proposed that memory retrieval is driven by multiple neural correlates, and research associates the sensitivity of the P6 ERP to indexing memory encoding, particularly across old/new paradigms^55,68^. A recent study by Andreau, et al.^69^ examined brain computations involved in visual stimuli target recognition and revealed a P6-like component related to recognition-based memory retrieval. In clinical trials, subjects diagnosed with mild AD demonstrated lower amplitudes and amplified latencies in ERPs associated with working memory, attention, and executive function^70,71^. These findings suggest complex relationships between working memory and other cognitive processes in the context of ERP components, particularly the late-positive P6^72,73^. The P6 has been shown to function as a binder for stimuli in memory recognition tasks involving previously encountered items^74^. Such discoveries suggest that P6 does far more than simply indexing memory events—it might integrate them as well. Even if P6 acts only as a computational partner in the course of multimodal target identification, P6 plays a relevant role in recognition-based memory retrieval^69^.

An investigation of scalp-recorded components of both the N4 and P6 ERPs enables a comprehensive analysis of some of the more observable and reliable measures of cognitive synchronicity and neural processing across broad cortical networks. These ERP components have been shown to be sensitive to AD-related changes and, as such, may be clinically useful markers. Researchers have long suggested that patients with even mild cognitive impairment have an increased risk of conversion to AD due to the presence of abnormalities in the N4 and P6 amplitudes^71,75,76^. This work demonstrated the predictive strength of N4/P6 anomaly analysis for predicting AD progression over time. As both ERP components are known to be sensitive to irregular declarative memory and semantic processing, N4 and P6 together might have significant clinical value in the neurological evaluation of episodic memory at the individual and group levels.

Using secondary data from a study conducted by Kilborn, et al.^77^ investigating ERP components as candidate biomarkers for AD, the present study sought to examine specific ERP measures for the purpose of distinguishing AD patients from a group of healthy age-matched controls. Using EEG analytical software, we assessed the performance of the N4 and P6 ERP components previously reported to be sensitive to AD in the early stages^55,71^. The amplitudes and latencies of the N4 and P6 ERPs were compared in a group of 63 mild, untreated AD patients and a control group of 73 healthy age-matched individuals. After calculating peak amplitudes and component latencies in their respective time windows and exploring main differences, ERP measurements were evaluated using analyses of variance (ANOVA) procedures for repeated measures. Post hoc comparisons revealed statistically significant effects for group and memory. Based on the literature presented, it was expected that healthy controls would outperform patients in peak amplitude and mean component latency across three parameters of memory when measured at optimal N4 (frontal) and P6 (parietal) locations. It was also predicted that the control group would exhibit neural cohesion through FPN integration during cross-modal tasks, thus demonstrating healthy cognitive functioning consistent with older healthy adults^10,49^.

## Method

### Participants

The participants included elderly patients and control individuals from the Kilborn, et al.^77^ study. The patients in the study were recruited from a specialist memory clinic. Ethical clearance was granted by the University of Glasgow College of Science and Engineering Ethics Committee, and written informed consent was obtained from every participant prior to study entry. The inclusion criteria consisted of referral to the Memory Clinic with the primary complaint of memory impairment, no pharmacological treatment beginning for AD or dementia, a score of ≥ 20 on the Mini-Mental State Examination (MMSE), age 65 years or older, a native English speaker of any ethnic origin, and met the NINCDS-ADRDA criteria for the diagnosis of possible AD^78^. Of the total number of patients (*n* = 83) who met these criteria, five were excluded due to technical problems. In addition, 15 patients were excluded based on a changed diagnosis at the one-year follow-up (vascular/mixed dementia or no dementia). A total of 76 healthy controls were recruited from the community. The data of three controls were rejected due to technical problems. These exclusions resulted in a final participant group (*n* = 136) consisting of 63 patients (32 males) and 73 controls (36 males). The mean age was 76.7 years (SD = 6.23) for the patient group and 73.3 years (SD = 5.92) for the control group. All participants had normal or corrected-to-normal vision and adequate hearing.

### Design

**Figure 1:**
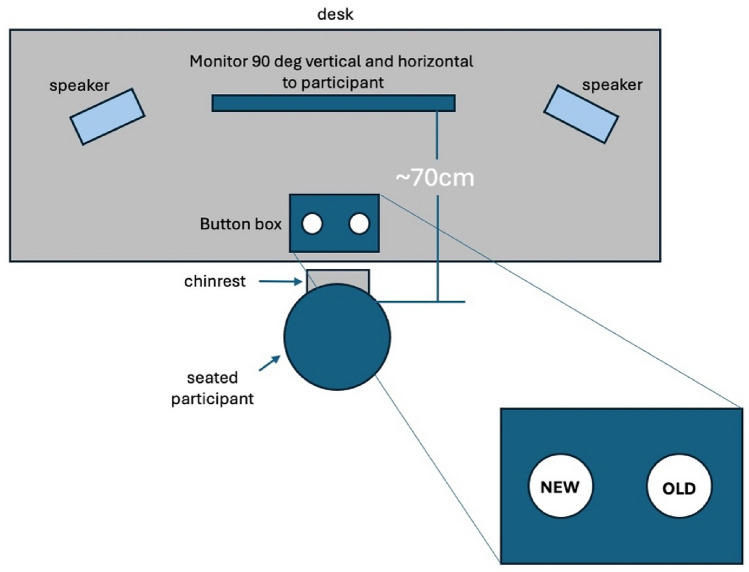
Schema of the task and experimental procedure, top looking down (not to scale).

Patients and controls were tested at four sites during the Kilborn, et al.^77^ study: Cognatec Research Centre Memory Clinic (Blackpool), Bradford Memory Clinic (Bradford), Memory Assessment Research Centre (Southampton), and Glasgow Memory Clinic (Clydebank). At the study visit, a brief background and the procedure were explained to the participant. The secondary EEG and behavioural data used for the present study were collected from participants while taking the Alzheimer’s Disease Evoked Potential Test (ADEPT) ^77^. The ADEPT consisted of associatively related stimulus pairs consisting of a synchronously presented picture and a spoken word presented on a computer screen (e.g., a picture of a train and the spoken word ‘tunnel’ such that some of these picture/word pairs were repeated after a delay. The task was designed to invoke hippocampal functioning because task performance requires (a) encoding and retrieval of short-term episodic memories and (b) processing and integration of cross-modal sensory input (i.e., visual and auditory)^45^. Colour pictures were presented in central vision on a black background, and the spoken words were presented through high-quality studio monitors. Participants were positioned at a viewing distance of approximately 70 cm; angles subtended by the visual stimuli were in each direction were 90° horizontally and 90° vertically. After either a short or long delay (6 or 39 intervening items, respectively), some stimulus pairs were presented for the second or third time, respectively. Participants were asked to decide whether each stimulus pair was presented for the first time (new item) or had been presented before (old item). Participants provided new or old judgement by pressing a button on a button box placed on the desk in front of them having the words “new” and “old” printed on the left and right buttons, respectively (Fig. 1). For the present study, memory events were sorted into different *bins* (i.e., categories) in preparation for group averaging.

A total of 270 items were presented: 110 in the new condition (i.e., items presented for the first time), 100 in the old/short condition (i.e., items presented a second time after 6 intervening items), and 60 in the old/long condition (i.e., items presented a second time after 39 intervening items). In each trial, a stimulus pair was presented for 3 s, after which the screen was turned black for 1 s. Participants had to give a response within 3 s following stimulus onset. The items were presented in two blocks to allow for a rest period. Responses were deemed accurate if they were made with the appropriate hand within a time frame of 100 to 3000 ms after the stimulus appeared. To assess response speed, we determined the 10% trimmed mean reaction times (RT). Errors were defined as incorrect responses made with the non-dominant hand, irrespective of the pace at which they were made. Furthermore, the discriminability index (d') was computed by subtracting the z-transforms for hits (i.e. properly identified old items) from the z-transforms for misses (i.e. mistakenly identified old items as new items). This index served as a measure of memory performance.

A sound test was carried out to assess the optimal sound level of the spoken words for each participant. The memory test was preceded by a training session of three practice blocks of ten trials each followed by the first test block. Following a short break, two more practice blocks and the final test block were completed. The total duration of the memory test was approximately 25 min. The study was performed in accordance with the Declaration of Helsinki^79^. The study used RStudio® as the primary statistical environment. EEG data were explored using EEGLAB, an interactive MATLAB® toolbox for processing continuous and event-related EEG data. ERP data were analysed using ERPLAB, an open-source MATLAB® package for analysing ERP data^80^. MATLAB® is a proprietary multiparadigm programming language and numeric computing environment developed by MathWorks®.

### EEG Recording and preprocessing

A 128-channel Geodesic Sensor Net was used to continually record the EEG^81^. The impedances were maintained at levels below 50 KΩ. The EEG signal was digitally sampled in real-time at a frequency of 250 Hz and subjected to band-pass filtering, restricting the frequency range to between 0.1 and 200 Hz. The ground electrode was placed near the vertex, specifically along the midline and anterior to Fz. The EEG data was divided into individual trial periods of 4000ms (with a 1000ms period before the shock) and filtered using the NetStation software, Electrical Geodesics Inc., Eugene, Oregon^82^. The approach involved the elimination of eye blink and movement artefacts from the EEG. Initially, the process of Independent Component Analysis (ICA) was performed using the EEGLAB software developed by Delorme and Makeig in 2004^83^. Subsequently, the temporal progression of each ICA component was cross-referenced with the time progression of each vertical (4 channels) and horizontal (2 channels) electrooculogram (EOG) channel. The ICA components that exhibited strong correlation with one or more EOG channel time courses, ranging from 7 to 25 components, were eliminated from the EEG data. Afterwards, epochs that had artefacts in one or more channels, as well as channels with noise, were identified and excluded from further research. The EEG data were low pass filtered at 30 Hz and average referenced. Ultimately, stimulus-locked ERPs were generated using a baseline of 100ms before the stimulus.

### Analysis

**Figure 2:**
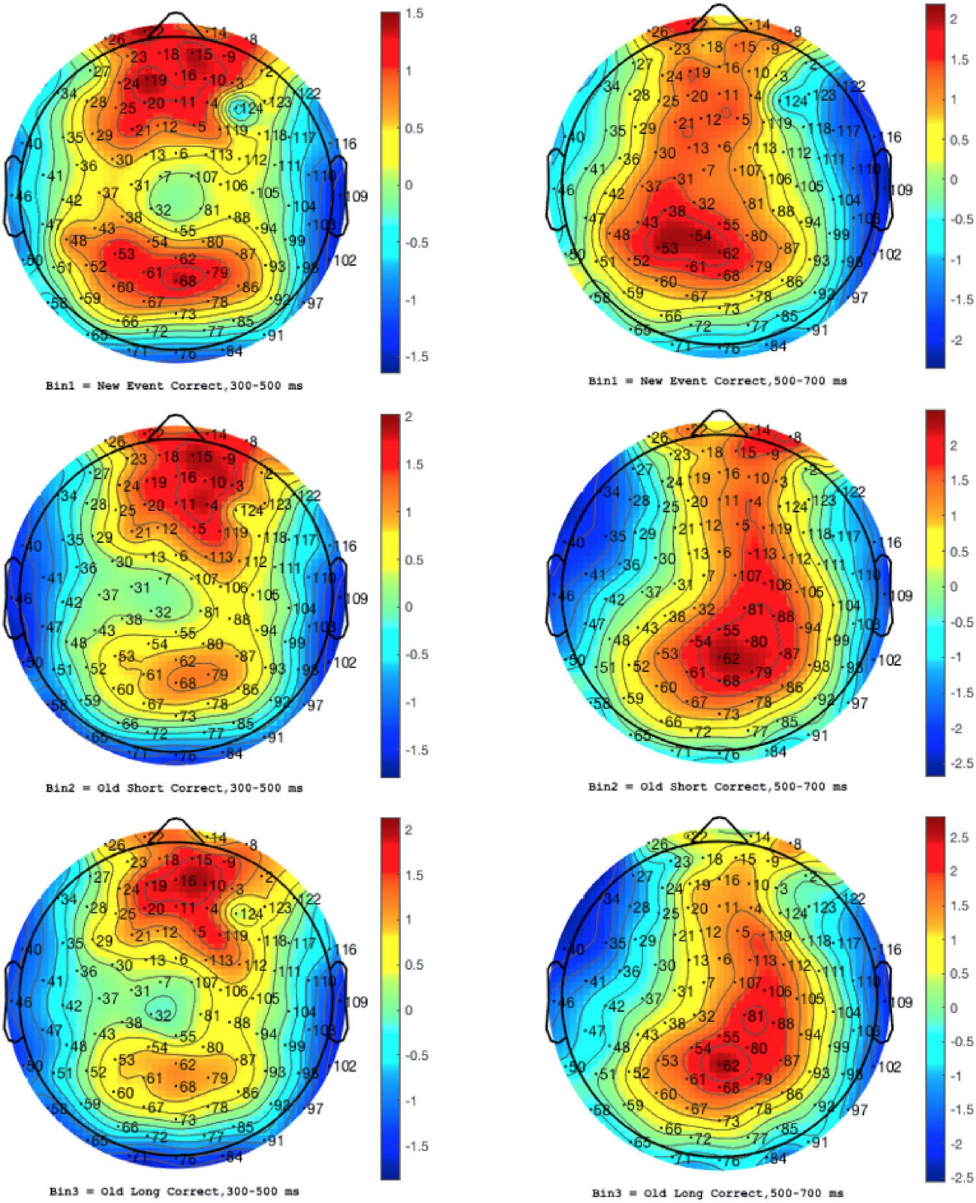
Grand average ERP scalp plots for (n = 73) healthy controls and correct answers, average across 300-500 ms and 500-700 ms latency windows.

The analysis was conducted using correct answers only, in this case, Category 1 through Category 3. The 300–500 and 500–700ms latency windows were selected to quantify the N4 and P6 measurements, respectively. Prior to analysis, channels 80 and 61 of the EEG sensor net were determined to be faulty through a full channel analysis, and the values were interpolated to allow for a fully accurate representation of the spatial data. Using scalp topography to visualize electric field activity, control grand mean amplitude values were examined to determine the most suitable reference channel clusters for N4 and P6 evaluation based on contour lines and positivity (Fig. 2). To analyse the secondary data, the present study used a 2 × 3 analysis of variance (ANOVA) procedure for repeated measures, with group as a between-subjects factor (controls vs. patients) and memory (new vs. old/short vs. old/long) as within-subject factors. The key independent variables were groups composed of healthy controls and AD patients. The key dependent variables consisted of N4/P6 ERP peak amplitudes in microvolts (µV) and N4/P6 ERP peak component latencies in milliseconds (ms). Memory was quantified by measuring participant RT to stimuli in milliseconds.

Amplitude values were translated to colour values, ranging from dark red for the most positive to dark blue for the most negative. For response strength, frontal channels E10, E15, E16, and E19 were selected to represent baseline N4 activity, while parietal channels E54, E55, E62, and E81 were chosen to represent baseline P6 activity.

## Results

To address the first hypothesis, ERP measures and memory were evaluated using 2 × 3 factorial analyses of variance (ANOVAs) for repeated measures, with group as a between-subjects factor (controls vs. patients) and memory (new vs. old/short vs. old/long) as within-subject factors. Using statistical modelling, the data across the dependent measures appeared normally and positively distributed, with the exception of latency measures, which demonstrated a positive platykurtic distribution likely due to under dispersed data. Three outliers involving amplitude were detected and removed from the raw data: two for a control participant in a P6 cluster channel and one for a patient in an N4 cluster channel. Outliers in this study were defined as points more extreme than Q1—1.5 * IQR or Q3 + 1.5 * IQR.

In first exploring the main differences, means and standard deviations were calculated for key dependent variables that consisted of the three conditions of memory measured as reaction time (RT in ms) and N4/P6 ERP peak amplitudes and component latencies. Simple main effect analyses showed significant effects on memory at the p < 0.001 level for all three conditions. The results indicated that the RT mean score for the control new group (M = 1085.62, SD = 165.79) was significantly lower than that for the patient new group (M = 1396.72, SD = 271.95) and that the RT mean score for the control old short group (M = 1015.31, SD = 146.15) was significantly lower than that for the patient old short group (M = 1334.97, SD = 270.32). Significantly lower RTs for old long conditions were also found in the controls (M = 988.13, SD = 161.15) and patients (M = 1297.45, SD = 276.79) (Table 1).

**Table 1:** Means and Standard Deviations: Control and Patient Memory (Reaction Time (RT)) in ms.

Measure	Control		Patient		*F*	Pr (> *F*)
	*M*	*SD*	*M*	*SD*		
Memory						
New	1085.62	165.79	1396.72	271.95	65.44	*p* < 0.001
Old Short	1015.31	146.15	1334.97	270.32	74.48	*p* < 0.001
Old Long	988.13	161.15	1297.45	276.79	64.06	*p* < 0.001

Simple main effect analysis also revealed a significant difference in ERP amplitudes (µV) at the p < 0.001 level between the two conditions [*F*(77, 37)]. The results indicated that the mean control N4 amplitude (M = 1.54, SD = 3.26) was significantly greater than the patient N4 amplitude (M = 0.58, SD = 3.08), and the mean control P6 amplitude (M = 1.98, SD = 2.58) was significantly greater than the patient P6 amplitude (M = 0.85, SD = 2.61). Additionally, there was a significant effect of control and patient condition on ERP component latencies at the p < 0.05 level for two conditions [*F*(6.7, 6.5)]. The results indicated that the mean score for control N4 latency (M = 404.74, SD = 57.50) was significantly lower than the patient N4 latency (M = 412.18, SD = 58.78) and that the mean score for control P6 latency (M = 598.00, SD = 60.20) was significantly lower than patient P6 latency (M = 605.66, SD = 61.25) (Table 2).

**Table 2:** Means and Standard Deviations: N4 and P6 Amplitudes in µV and Component Latency in ms.

Measure	Control		Patient		*F*	Pr (> *F*)
	*M*	*SD*	*M*	*SD*		
N4 Amplitude	1.54	3.26	0.58	3.08	36.89	*p* < 0.001
N4 Latency	404.74	57.50	412.18	58.78	6.65	*p* < 0.05
P6 Amplitude	1.98	2.58	0.85	2.61	76.94	*p* < 0.001
P6 Latency	598.00	60.20	605.66	61.25	6.53	*p* < 0.05

**Figure 3:**
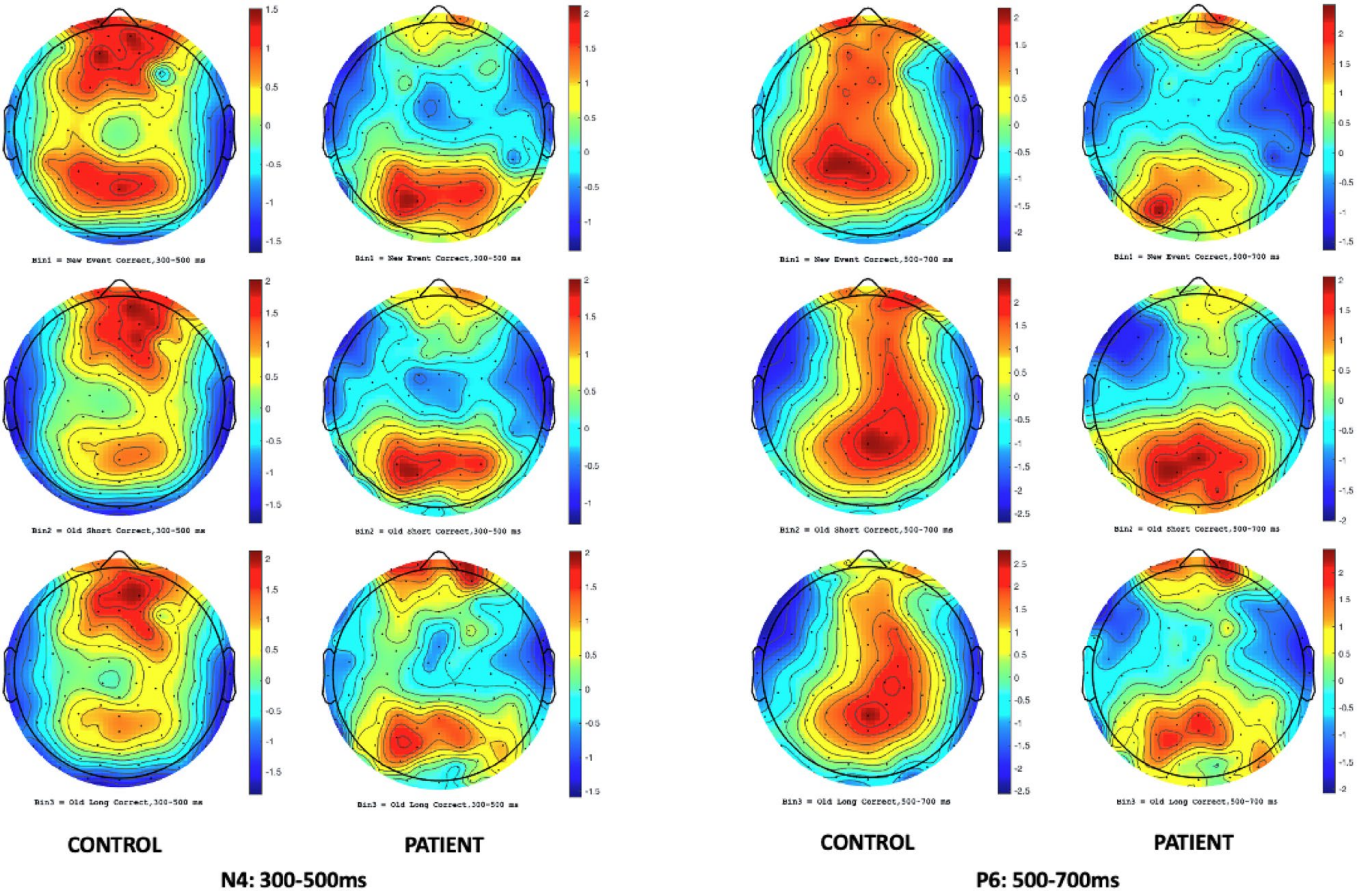
Frontal/parietal mean amplitude scalp map comparison (n = 73 control; n = 63 patient; N4 and P6 latency windows).

To enhance the main effect, the plotting of ERP scalp maps afforded a visual support for the initial conclusions, as EEG topography localized and differentiated N4/P6 amplitudes in the frontal and parietal regions of controls and patients within their respective component latency windows (Fig. 3). Multiple alternatives exist for ascertaining the scale of the topographic maps. Choosing the Max–Min option, the programme will automatically assign a scale to each bin based on the lowest and highest voltages discovered in that bin. For example, the selected scale might range from -4 µV to + 9 µV. Choosing Abs-Max, the algorithm identifies the minimum and maximum values and generates a symmetrical scale based on whichever number has a larger absolute magnitude. Our study opted for the Max–Min approach for enhanced mapping precision.

To assess the range of group differences, a two-way ANOVA was conducted to examine the variation of groups on the three [correct] conditions of RTs. No statistically significant group effects or evidence of interaction were identified in this analysis across new, old/short, or old/long memory conditions (Table 3).

**Table 3:** Factorial ANOVA Results for Within-Subjects Effects: Reaction Times (RTs).

Factor	Sum of Squares	df	Mean Square	*F*	Pr (> *F*)
Group	62.5	1	62.53	4.07	*p* < 0.05
New	0.4	1	0.43	0.03	0.87
Group * new	3.7	1	3.67	0.24	0.63
Group	62.5	1	62.53	4.09	*p* < 0.05
Old short	0.3	1	0.31	0.02	0.89
Group * old short	16.4	1	16.40	1.07	0.30
Group	62.5	1	62.53	4.07	*p* < 0.05
Old long	2.2	1	2.25	0.15	0.70
Group * old long	1.2	1	1.23	0.08	0.78

Finally, two-way ANOVA was conducted to evaluate the effect of group on ERPs (µV) (Table 4). We found a statistically significant difference in N4 amplitude [*F*(1) = 32.18, p < 0.001] and N4 latency [*F*(1) = 31.41, p < 0.001] between the groups, although linear models showed no interaction between these factors. A Tukey HSD post hoc test revealed significant pairwise differences between patients and controls (-0.96 in N4 amplitude and 7.44 in N4 latency). We also found a statistically significant difference in P6 amplitude [*F*(1) = 11.12 p < 0.001] and P6 latency [*F*(1) = 11.06, p < 0.001] between the groups, and linear models showed no interaction between these factors. A Tukey HSD post hoc test revealed significant pairwise differences between patients and controls (-1.13 in P6 amplitude and 7.70 in P6 latency).

**Table 4:** Factorial ANOVA Results for Within-Subjects Effects: N4/P6 ERP Amplitudes and Latencies.

Factor	Sum of Squares	df	Mean Square	*F*	Pr (> *F*)
Group	22,436	1	22,436	6.77	*p* < 0.01
N4 amplitude	476	1	476	0.14	0.70
Group * N4 amplitude	106,619	1	106,619	32.18	*p* < 0.001
Group	372	1	372.3	37.56	*p* < 0.001
N4 Latency	1	1	1.4	0.14	0.71
Group * N4 latency	311	1	311.3	31.41	*p* < 0.001
Group	24,046	1	24,046	6.58	*p* < 0.05
P6 amplitude	9093	1	9093	2.49	0.12
Group * P6 amplitude	40,656	1	40,656	11.12	*p* < 0.001
Group	519	1	518.6	77.49	*p* < 0.001
P6 Latency	17	1	16.6	2.49	0.12
Group * P6 Latency	74	1	74.0	11.06	*p* < 0.001

## Discussion

Using a cross-modal associative memory test in combination with high-density EEG, this study examined electrophysiological cognitive measurements that might serve as candidate functional biomarkers of AD. By employing scalp topography to visualize electric field activity, it was possible to select the best reference channels for exploring the frontal and parietal regions of participants. While no statistically significant group effects or interactions were identified across the three memory conditions, our results revealed statistically significant group effects between controls and patients in N4/P6 amplitudes and latencies during cross-modal memory tasks, although there was no evidence of interaction. Our findings also support that N4 ERP anomalies are potentially more effective than their P6 counterparts for use as possible candidate AD biomarkers. Furthermore, we visually established how healthy control participants potentially exhibit neural cohesion through FPN shifting during cross-modal tasks, whereas AD patients do not. These findings are consistent with research proposing that N4/P6 ERPs together might serve as potentially useful biomarkers for AD across a variety of cognitive functions^75,84–86^. Like the findings of the Olichney, et al.^71^ study, which investigated ERP biomarkers of progression and conversion to dementia in individuals with mild cognitive impairment (MCI), our findings reinforce the theory that abnormalities in N4 and P6 together might be associated with the risk of conversion to mild AD. Several studies investigating N4 effects as a result of manipulating congruity revealed reduced N4 amplitudes and slower component latencies when comparing AD-diagnosed individuals with healthy controls^52,87,88^. Research also supports that significant abnormalities in late-positive P6 in mild AD patients continue to result in reduced sensitivity, particularly in studies involving stimuli repetition^55,89^. Taken together, N4 and P6 potentially serve as suitable co-indices for memory encoding and retrieval processes during the course of episodic and declarative memory.

It is important to highlight that the present study likewise recognized the significance of reaction time slowing at the individual level as a potential indicator of AD; however, there is little empirical evidence in this paper to support the effectiveness of RT as a biomarker, nor was this the principal intent. There are environmental and physiological variables internal and external to this study that might influence participant reaction times/memories. Reaction times were relevant and useful for channel categorization and general observation alone and, as such, were not the focus of this discussion. Taken together, our results were consistent with the hypothesis that healthy controls would outperform patients in terms of peak amplitude and mean component latency across three parameters of memory when measured at the optimum N4 (frontal) and P6 (parietal) locations. It was also predicted that the control group would exhibit neural cohesion through FPN integration during cross-modal tasks, thus demonstrating healthy cognitive functioning consistent with that of healthy adults^10,25,26,90^. A comparative evaluation of topographic scalp mapping and EEG waveform plotting was conducted on healthy controls and mildly untreated AD patients to address our second hypothesis.

### Cortical activity shift

**Figure 4:**
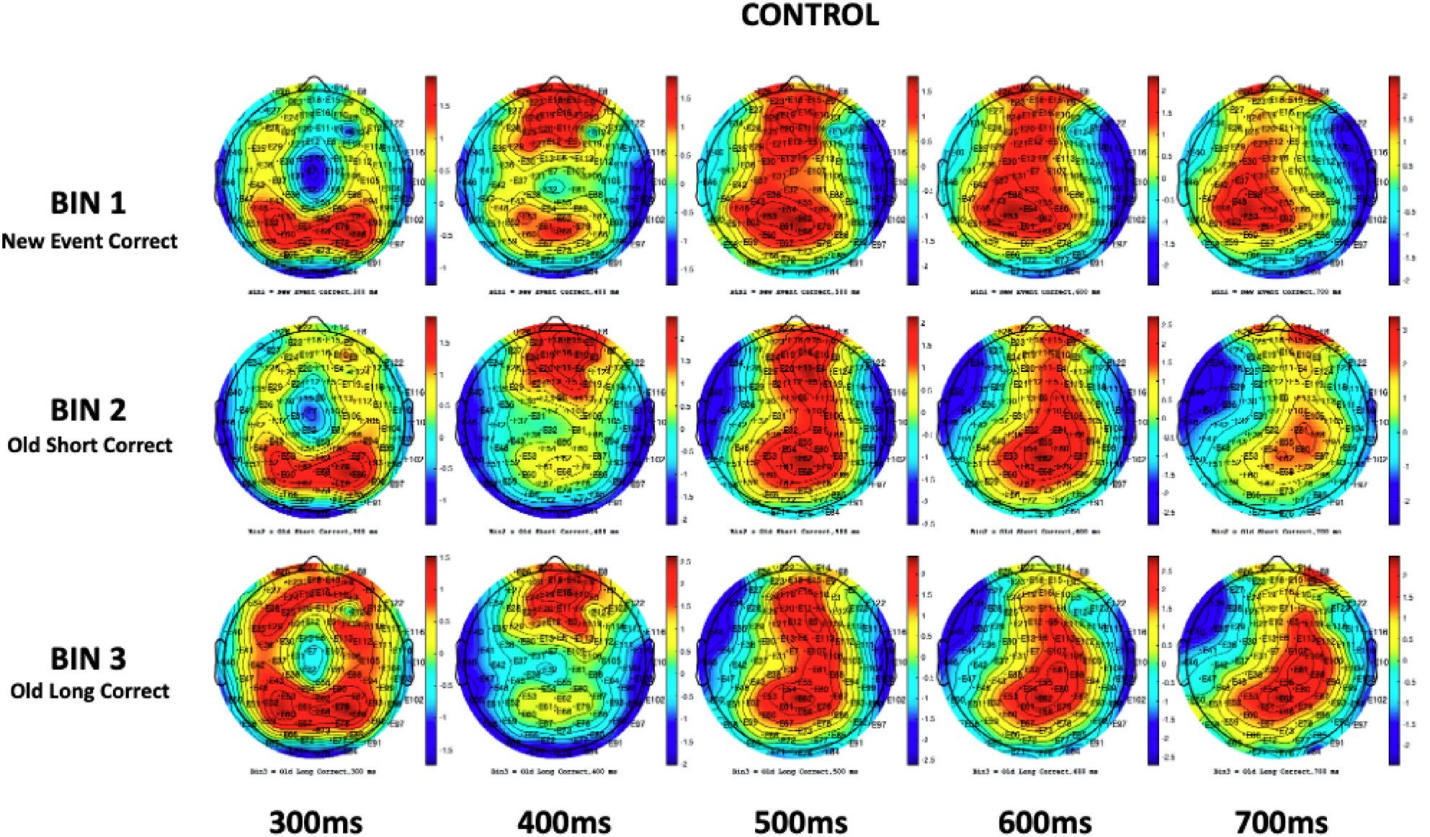
Control (n = 73) scalp maps: Bin 1 – 3. Grand mean amplitudes at 300 ms, 400 ms, 500 ms, 600 ms, and 700 ms.

**Figure 5:**
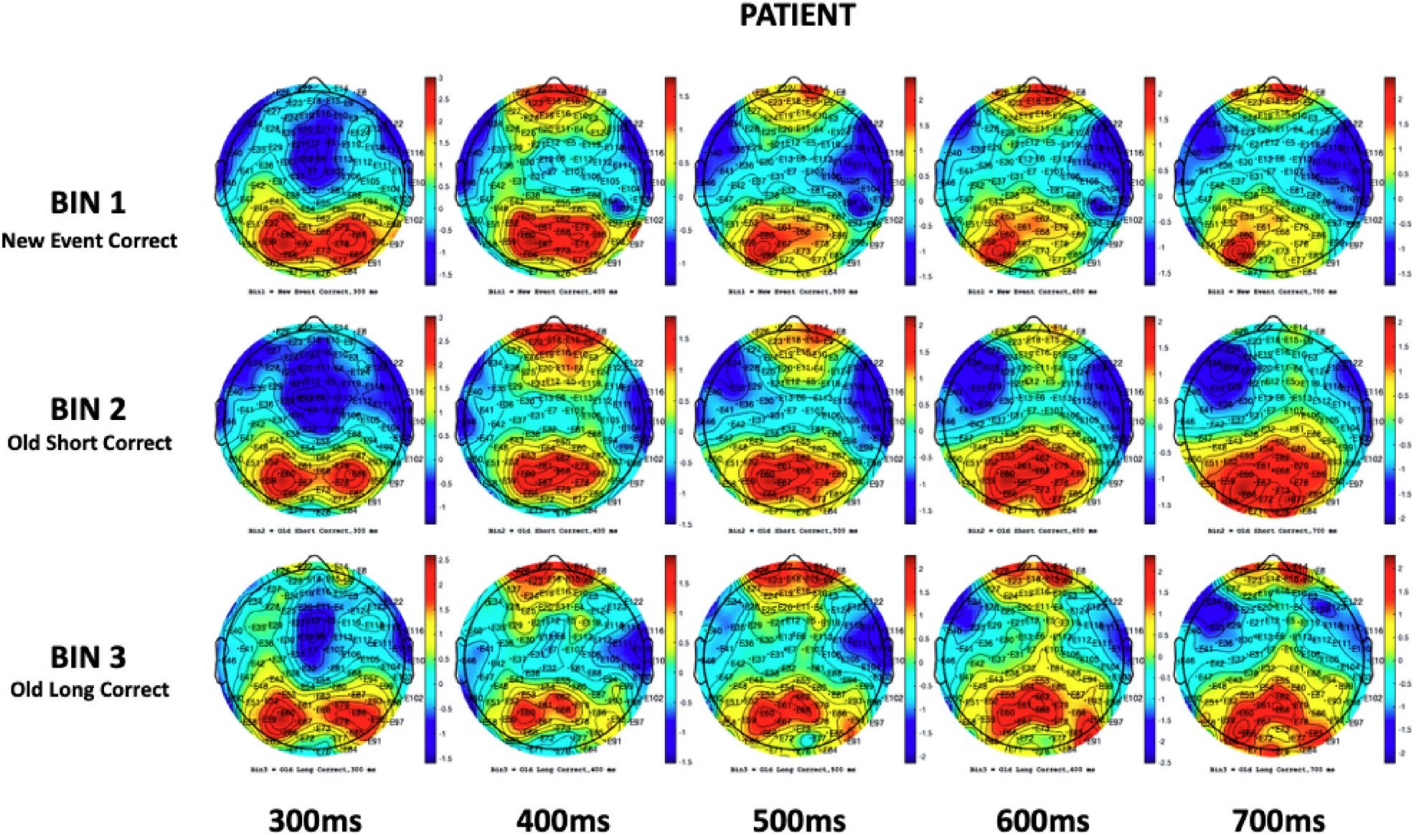
Patient (n = 63) scalp maps: Bin 1 – 3. Grand mean amplitudes at 300 ms, 400 ms, 500 ms, 600 ms, and 700 ms.

To demonstrate the frontoparietal shift notable by Spreng and Turner^49^, grand mean amplitudes of the scalp topography between the 300ms and 700ms component latencies were captured during the cross-modal tasks for healthy controls as a baseline reference (Fig. 4). After conducting a visual inspection at a stimulus onset of ~ 300ms, there was noticeable positive activity in the parietal regions of participants in all three memory conditions. Towards the peak of N4 activity, an apparent reliance on the frontal cortex materializes, with little positivity in the parietal region, particularly during old/short and old/long memory conditions. This activity is suggestive of both the Buckner^23^ work proposing predominant frontal recruitment during problem solving as a sign of normal aging and the dual-process model put forward by Rugg and Yonelinas^91^ suggesting that distinct frontoparietal neural mechanisms support recollection and familiarity. Additionally, while dissimilar N4 and P6 topographic effects have been presented between healthy aging adults and AD patients in the past^52,73^, few studies have visually demonstrated the systematic shift from a predominantly frontal reliance to parietal reliance in the encoding and recall of episodic memories. The resulting topographical illustration of FPN connectivity pre-to-post stimulus is also consistent with theories suggesting interplay between the hippocampal region and prefrontal cortex in the assimilation of new memories^92^. Between 500 and 600ms, there was a dispersal of positive electrophysical activity between the frontal and parietal cortices, with P6 ERP activity beginning to peak at 600ms. At 700ms, cortical activity in the control group almost exclusively shifted to the parietal lobe. Notwithstanding cortical functions, one observation becomes apparent; there is a dearth of frontoparietal connectivity/shifting in mild untreated AD patients in the same time window across similar memory paradigms when compared to healthy controls (Fig. 5).

**Figure 6:**
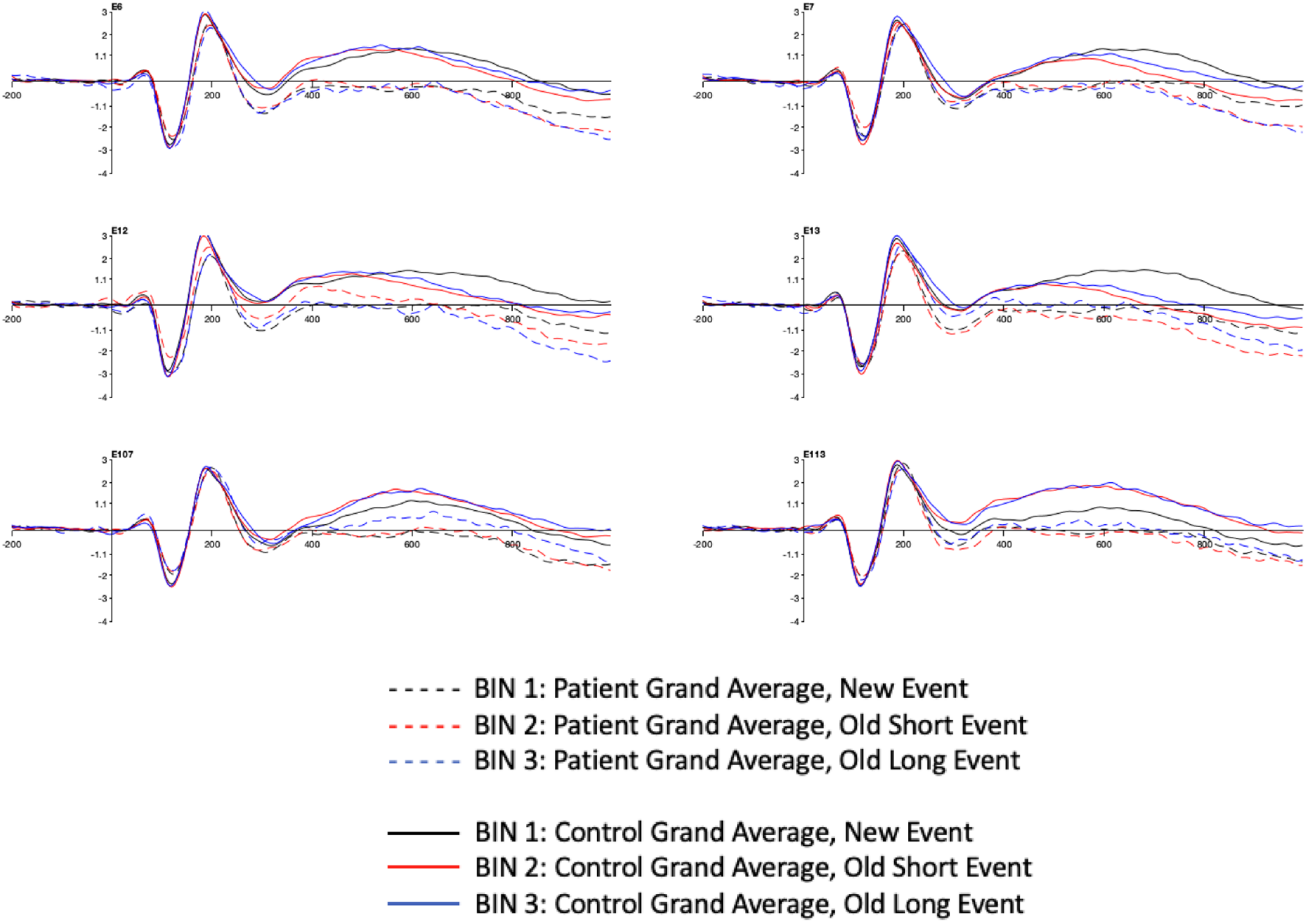
Mid-cortical Comparative Waveforms: Control (n = 73) and Patient (n = 63) Grand Averages; N4 and P6 ERPs; 30 Hz, low pass filtered and averaged.

Similar to identifying and selecting the reference channels for our baseline ERP activity based on positivity, effectively studying variances where activity is present and scalp topography affords us a clear picture of where to explore reference channel clusters where activity is absent but should be present. Channels E6, E7, E12, E13, E107, and E113 were chosen to function as mid-cortical neural pathway checkpoints between the frontal and parietal regions of participants in each group. Waveforms were chosen as the medium for comparative analysis to define and contrast locations, amplitudes, and frequencies of electrophysiological brain activity. More importantly, waveforms offered further validation of our topographical ERP measurements. Attenuation of N4 and P6 ERP amplitudes at the midpoint reference channel locations in the three memory conditions was clear in the AD patients compared to the waveforms of healthy controls (Fig. 6).

In summary, the present study demonstrated a significant, measurable decrease in N4/P6 ERP amplitude activity accompanied by a subsequent increase in N4/P6 ERP component latencies in mild untreated AD patients. The study also revealed the occurrence of neural cohesion through FPN shifting during cross-modal tasks in healthy adults. The usefulness of EEG scalp topography is established by selecting reference EEG channels, conducting comparative analyses between ERPs, and visualizing functionally integrated relationships between dispersed brain regions. By way of neural pathway checkpointing, we see that an evaluation of cortical relationships can be performed not only through what is present but also through what is not. Considering the results of the literature review and the results of our study, we identified a number of explanations for the lack of electrophysical activity in specific brain regions of AD patients. Healthy cognitive functioning during the course of memory at least in part appears contingent on successful frontoparietal connectivity. A paper by Delbeuck, et al.^93^ reviewed evidence from a number of electrophysiological and neuroimaging studies and explored the effect of cerebral disconnections on cognitive functioning. Researchers concluded that these cessations stemmed from disruptions involving neural impulses moving toward and away from different areas of the brain. More recent findings reveal weak connections between cortical regions as a result of inconsistent brain wave oscillations, structural brain changes, and fibre connectivity density^94–96^. In the context of these analyses, the question remains whether structural change precedes functional connectivity or vice versa. Our present study faces a similar dilemma. As a combination of ERP amplitude, latency, and scalp topography provides us with indications of where these disconnections might occur, they fall short in suggesting to what degree AD performance on the cross-modal memory task is a by-product of cortical disengagement or diminished ERP performance.

Promising techniques involving transcranial magnetic stimulation (TMS) applied in AD research have been conducted over the past decade. Like EEGs, TMS is a safe, non-invasive, and painless method widely used to study brain functions^97^. By using brief, high-intensity magnetic fields to induce currents and depolarize neurons in various areas of the brain, TMS shows potential as a targeted strategy to modulate electrical activity in the brain^98^. Originally used for cortical exploration and peripheral nerve excitation, current research includes an expanded range of TMSs to include localized activation/deactivation of specific areas within the cerebral cortex^99^. Conceptually speaking, Bergmann^100^ asserts that TMS applied in this fashion creates open and closed-loop electrical systems for analysis where the brain itself might inform input/output. Applied to our present dilemma, a recent study by Esposito, et al.^101^ captured the usefulness of an integrated EEG/TMS methodology for studying brain connectivity; notably, the credible advantage of combining different analytical methods is that it produces a single more complex instrument while systematically reducing variability in the data by offering more information. In essence, the gap between function and structure is significantly reduced by integrative neuroscience. Combined approaches also allow us to better characterize the relationships between brain structure and neural response, ultimately enhancing our understanding of cortical connectivity, or lack thereof, in patients with AD.

### Limitations

Limitations of this study include its relatively small sample size. A larger participant sample would provide more accurate mean values, which are critical when considering clinical prediction models^102^. As multichannel EEG methods exceed sparse array systems in spatial resolution and signal quality (e.g., 128 vs. 10/20 channel systems), source signals measured at the scalp nevertheless have limitations in the exact detection and localization of some neuropathologies^103,104^. The use of higher-density EEG arrays might be useful in similar research to obtain more detailed electrophysiological measurements of brain activity or absence thereof. As a single neuroimaging modality was used for this analysis, the integration of EEG signals with other functional/structural neuroimaging modalities, including but not limited to TMS, fMRI, structural magnetic resonance imaging (sMRI), magnetoencephalography (MEG), and diffusion tensor imaging (DTI), will likely enhance the accuracy, detection, and differential diagnosis of cognitive impairment in aging adults ^105–109^. Finally, performance tests involving computers, regardless of assessed operational ease, might be influenced by computer experience, especially considering the ages of some participants in this study and the year in which they were conducted^110^. Bozionelos and Di Giacomo demonstrated that intimidation and anxiety can affect the performance of individuals with limited exposure to technology^111,112^. Alternative interface applications for elderly participants might be considered for use in future studies, particularly those using reaction time as a measure of memory^113–115^.

## Conclusion

High-density EEG methods continue to show promise as an accurate, economical, non-invasive approach for measuring and categorizing the electrical potentials of brains, particularly those associated with AD. By merging scalp topography analysis with N4/P6 ERP amplitude and latency measurements, the present study demonstrates how healthy adult cognition during cross-modal memory events is dependent on the activation and performance of distinct ERPs while systematically being sustained by apparent functionally dissociable processes spanning the frontoparietal network. Our results revealed a distinct N4/P6 ERP amplitude attenuation and subsequent increase in component latency in mild untreated AD patients during cross-modal memory tasks, while systematically revealing statistically significant group effects on N4/P6 amplitudes and latencies in controls and patients who made old/new recognition judgments. Our findings provide further evidence that N4 ERP anomalies are potentially more effective than their P6 counterparts for use as possible candidate AD biomarkers. Using EEG scalp topography, we visually demonstrated how and approximately where healthy control participants demonstrated neural cohesion through FPN shifting during cross-modal tasks, whereas the AD patients did not. By better defining EEG biomarkers, it becomes more tangible to assess their translational value for the discovery of new drugs and treatments. We hope this study will be able to inform future research on the combined analysis of N4/P6 ERPs in AD memory research, particularly in the context of multimodal stimuli; and expand the dialogue on FPN integration in the course of executive functioning.

### Note

While EEG topographical analysis has become popular in the clinical setting, we are thoughtful of studies arguing that a reliance on high-contrast colour mapping be approached with caution, as it potentially misleads the viewer by introducing artefacts to visualization, thus obscuring information by limiting apparent changes at colour boundaries^116,117^. The result is an impression of substantial changes when the actual differences may be quite small. The present study used high-contrast colour mapping for illustrative purposes and verified reference channel selection via lower-contrast gradient maps and associated scales during analysis. Even as modern EEG software provides researchers with a more accurate scalp topography interface, the use of alternate colour maps might effectively translate better to printed media in addition to making topographical data inclusive to colourblind viewers.

### Supplementary Information

Supplementary Information 1.

Supplementary Information 2.

Supplementary Information 3.

Supplementary Information 4.

## Data Availability

Data for this study is provided in the supplemental information files.

## References

[1] Du X, Wang X, Geng M (2018). Alzheimer’s disease hypothesis and related therapies. Transl. Neurodegener..

[2] Ashraf A, So P-W (2020). Spotlight on ferroptosis: Iron-dependent cell death in Alzheimer’s disease. Front. Aging Neurosci..

[3] Theofilas P (2018). Probing the correlation of neuronal loss, neurofibrillary tangles, and cell death markers across the Alzheimer's disease Braak stages: A quantitative study in humans. Neurobiol. Aging.

[4] Zubair M (2019). Biological Diagnostic and Therapeutic Advances in Alzheimer's Disease.

[5] Gouras GK (2019). Aging, metabolism, synaptic activity and Aβ in Alzheimer’s disease. Front. Aging Neurosci..

[6] Al-Qazzaz NK (2014). Role of EEG as biomarker in the early detection and classification of dementia. Sci. World J..

[7] Peters S, Van Duijvenvoorde ACK, Koolschijn PCMP, Crone EA (2016). Longitudinal development of frontoparietal activity during feedback learning: Contributions of age, performance, working memory and cortical thickness. Dev. Cogn. Neurosci..

[8] Bennys K, Rondouin G, Vergnes C, Touchon J (2001). Diagnostic value of quantitative EEG in Alzheimer’s disease. Neurophysiol. Clin./Clin. Neurophysiol..

[9] Xiang H-D, Fonteijn HM, Norris DG, Hagoort P (2009). Topographical Functional Connectivity Pattern in the Perisylvian Language Networks. Cereb. Cortex.

[10] Ray KL (2020). Dynamic reorganization of the frontal parietal network during cognitive control and episodic memory. Cogn. Affect. Behav. Neurosci..

[11] Horn H (2012). Semantic network disconnection in formal thought disorder. Neuropsychobiology.

[12] Blackwood DH, Muir WJ (1990). Cognitive brain potentials and their application. Br. J. Psychiatry Suppl..

[13] Nunez PL, Srinivasan R (2006). Electric fields of the brain: the neurophysics of EEG.

[14] Babiloni C (2020). What electrophysiology tells us about Alzheimer's disease: a window into the synchronization and connectivity of brain neurons. Neurobiol. Aging.

[15] Curran T, DeBuse C, Leynes PA (2007). Conflict and criterion setting in recognition memory. J. Exp. Psychol. Learn. Mem. Cogn..

[16] Tulving E (2002). Episodic memory: From mind to brain. Annu. Rev. Psychol..

[17] Li Y, Seger C, Chen Q, Mo L (2020). Left inferior frontal gyrus integrates multisensory information in category learning. Cereb. Cortex.

[18] Nee DE (2012). A Meta-analysis of Executive Components of Working Memory. Cereb. Cortex.

[19] Li L, Gratton C, Fabiani M, Knight RT (2013). Age-related frontoparietal changes during the control of bottom-up and top-down attention: an ERP study. Neurobiol. Aging.

[20] Müller NG, Knight RT (2002). Age-related changes in fronto-parietal networks during spatial memory: an ERP study. Cogn. Brain Res..

[21] Pehlivanoglu D, Duarte A, Verhaeghen P (2020). Multiple identity tracking strategies vary by age: An ERP study. Neuropsychologia.

[22] Friedman D, Kazmerski V, Fabiani M (1997). An overview of age-related changes in the scalp distribution of P3b. Electroencephal. Clin. Neurophysiol./Evok. Potent. Sect..

[23] Buckner RL (2004). Memory and Executive function in aging and AD: Multiple factors that cause decline and reserve factors that compensate. Neuron.

[24] Posner MI, Dehaene S (1994). Attentional networks. Trends Neurosci..

[25] Zanto TP, Gazzaley A (2013). Fronto-parietal network: flexible hub of cognitive control. Trends Cogn. Sci..

[26] Chadick JZ, Gazzaley A (2011). Differential coupling of visual cortex with default or frontal-parietal network based on goals. Nat. Neurosci..

[27] Fischer M, Moscovitch M, Alain C (2020). A systematic review and meta-analysis of memory-guided attention: Frontal and parietal activation suggests involvement of fronto-parietal networks. Wiley Interdiscipl. Rev. Cogn. Sci..

[28] Thakral PP, Madore KP, Schacter DL (2017). A role for the left angular gyrus in episodic simulation and memory. J. Neurosci..

[29] Tibon R, Fuhrmann D, Levy DA, Simons JS, Henson RN (2019). Multimodal integration and vividness in the angular gyrus during episodic encoding and retrieval. J. Neurosci..

[30] van der Linden M, Berkers RMWJ, Morris RGM, Fernández G (2017). Angular gyrus involvement at encoding and retrieval is associated with durable but less specific memories. J. Neurosci..

[31] Valdois S, Lassus-Sangosse D, Lallier M, Moreaud O, Pisella L (2019). What bilateral damage of the superior parietal lobes tells us about visual attention disorders in developmental dyslexia. Neuropsychologia.

[32] Rushworth MFS, Walton ME, Kennerley SW, Bannerman DM (2004). Action sets and decisions in the medial frontal cortex. Trends Cogn. Sci..

[33] Kamiński J (2017). Persistently active neurons in human medial frontal and medial temporal lobe support working memory. Nat. Neurosci..

[34] Owens MM, Duda B, Sweet LH, MacKillop J (2018). Distinct functional and structural neural underpinnings of working memory. NeuroImage.

[35] Gruber SA (2017). Decreased Cingulate Cortex activation during cognitive control processing in bipolar disorder. J. Affect. Disord..

[36] Tolomeo S (2016). A causal role for the anterior mid-cingulate cortex in negative affect and cognitive control. Brain.

[37] Tulving E, Thomson DM (1973). Encoding specificity and retrieval processes in episodic memory. Psychol. Rev..

[38] Stopford CL, Thompson JC, Neary D, Richardson AMT, Snowden JS (2012). Working memory, attention, and executive function in Alzheimer’s disease and frontotemporal dementia. Cortex.

[39] Karrasch M (2006). Brain oscillatory responses to an auditory-verbal working memory task in mild cognitive impairment and Alzheimer's disease. Int. J. Psychophysiol..

[40] Maruszak A, Thuret S (2014). Why looking at the whole hippocampus is not enough-a critical role for anteroposterior axis, subfield and activation analyses to enhance predictive value of hippocampal changes for Alzheimer's disease diagnosis. Front. Cell. Neurosci..

[41] Mu Y, Gage FH (2011). Adult hippocampal neurogenesis and its role in Alzheimer's disease. Mol. Neurodegener..

[42] McClelland JL, McNaughton BL, O'Reilly RC (1995). Why there are complementary learning systems in the hippocampus and neocortex: insights from the successes and failures of connectionist models of learning and memory. Psychol. Rev..

[43] Kumaran D, Hassabis D, McClelland JL (2016). What learning systems do intelligent agents need? Complementary learning systems theory updated. Trends Cogn. Sci..

[44] Shastri L (2002). Episodic memory and cortico–hippocampal interactions. Trends Cogn. Sci..

[45] Gottlieb LJ, Uncapher MR, Rugg MD (2010). Dissociation of the neural correlates of visual and auditory contextual encoding. Neuropsychologia.

[46] Barker GRI, Warburton EC (2020). Putting objects in context: A prefrontal–hippocampal–perirhinal cortex network. Brain Neurosci. Adv..

[47] Eichenbaum H (2017). Prefrontal–hippocampal interactions in episodic memory. Nat. Rev. Neurosci..

[48] Miller EK, Cohen JD (2001). An integrative theory of prefrontal cortex function. Annu. Rev. Neurosci..

[49] Spreng RN, Turner GR (2019). The shifting architecture of cognition and brain function in older adulthood. Perspect. Psychol. Sci..

[50] Van Buuren M, Wagner IC, Fernández G (2019). Functional network interactions at rest underlie individual differences in memory ability. Learn. Memory.

[51] Cheyette SJ, Plaut DC (2017). Modeling the N400 ERP component as transient semantic over-activation within a neural network model of word comprehension. Cognition.

[52] Auchterlonie, S., Phillips, N. A. & Chertkow, H. Behavioral and electrical brain measures of semantic priming in patients with Alzheimer's disease: implications for access failure versus deterioration hypotheses. *Brain Cogn.* (2002). 12030448

[53] Grieder M (2013). Correlation between topographic N400 anomalies and reduced cerebral blood flow in the anterior temporal lobes of patients with dementia. J. Alzheimer's Dis..

[54] Wolk DA (2005). Patients with mild Alzheimer's disease attribute conceptual fluency to prior experience. Neuropsychologia.

[55] Olichney (2006). Absent event-related potential (ERP) word repetition effects in mild Alzheimer's disease. Clin. Neurophysiol..

[56] Kutas M, Federmeier KD (2000). Electrophysiology reveals semantic memory use in language comprehension. Trends Cogn. Sci..

[57] Nigam A, Hoffman JE, Simons RF (1992). N400 to Semantically Anomalous Pictures and Words. J. Cogn. Neurosci..

[58] Rugg MD, Curran T (2007). Event-related potentials and recognition memory. Trends Cogn. Sci..

[59] Vilberg KL, Rugg MD (2008). Memory retrieval and the parietal cortex: A review of evidence from a dual-process perspective. Neuropsychologia.

[60] Atkinson RC, Juola JF (1974). Search and decision processes in recognition memory.

[61] Tulving E (1985). Memory and consciousness. Can. Psychol./Psychol. Can..

[62] Curran T (2000). Brain potentials of recollection and familiarity. Memory Cogn..

[63] Paller KA, Kutas M (1992). Brain potentials during memory retrieval provide neurophysiological support for the distinction between conscious recollection and priming. J. Cogn. Neurosci..

[64] Voss JL, Federmeier KD (2011). FN400 potentials are functionally identical to N400 potentials and reflect semantic processing during recognition testing. Psychophysiology.

[65] van Herten M, Kolk HHJ, Chwilla DJ (2005). An ERP study of P600 effects elicited by semantic anomalies. Cogn. Brain Res..

[66] Shen W, Fiori-Duharcourt N, Isel F (2016). Functional significance of the semantic P600: Evidence from the event-related brain potential source localization. NeuroReport.

[67] Schloerscheidt AM, Rugg MD (2004). The impact of change in stimulus format on the electrophysiological indices of recognition. Neuropsychologia.

[68] Burkhardt P (2007). The P600 reflects cost of new information in discourse memory. Neuroreport.

[69] Andreau JM, Idesis SA, Iorio AA (2020). Unraveling the electrophysiological activity behind recognition memory. J. Psychophysiol..

[70] Cecchi M (2015). A clinical trial to validate event-related potential markers of Alzheimer's disease in outpatient settings. Alzheimer's Dementia Diagn. Assess. Dis. Monitor..

[71] Olichney (2008). Patients with MCI and N400 or P600 abnormalities are at very high risk for conversion to dementia. Neurology.

[72] O'Rourke, P. The interaction of different working memory mechanisms and sentence processing: A study of the P600. In *Proc. of the Annual Meeting of the Cognitive Science Society* **35**, 35(2013).

[73] Guillem F, N'Kaoua B, Rougier A, Claverie B (1995). Intracranial topography of event-related potentials (N400/P600) elicited during a continuous recognition memory task. Psychophysiology.

[74] Xia J (2020). Event-related potential and EEG oscillatory predictors of verbal memory in mild cognitive impairment. Brain Commun..

[75] Olichney (2002). Abnormal verbal event related potentials in mild cognitive impairment and incipient Alzheimer's disease. J. Neurol. Neurosurg. Psychiatry.

[76] Kutas M, Federmeier KD (2009). N400. Scholarpedia.

[77] Kilborn K (2009). Cognitive event related potentials as functional biomarkers in Alzheimer's disease. Alzheimer's Dementia.

[78] McKhann G (1984). Clinical diagnosis of Alzheimer's disease: report of the NINCDS-ADRDA Work Group under the auspices of department of health and human services task force on Alzheimer's disease. Neurology.

[79] World Medical, A. Declaration of Helsinki, ethical principles for medical research involving human subjects. *52 nd WMA General Assembly, Edinburgh, Scotland* (2000).

[80] Lopez-Calderon J, Luck SJ (2014). ERPLAB: An open-source toolbox for the analysis of event-related potentials. Front. Hum. Neurosci..

[81] Tucker DM (1993). Spatial sampling of head electrical fields: the geodesic sensor net. Electroencephal. Clin. Neurophysiol..

[82] Michel, C. M. *et al.* EEG source imaging. *Clin. neurophysiol.* **115**(10), 2195–2222 (2004). 10.1016/j.clinph.2004.06.001 15351361

[83] Delorme A, Makeig S (2004). EEGLAB: an open source toolbox for analysis of single-trial EEG dynamics including independent component analysis. J. Neurosci. Methods.

[84] Jackson, C. E., & Snyder, P. J. Electroencephalography and event-related potentials as biomarkers of mild cognitive impairment and mild Alzheimer’s disease. *Alzheimer's & Dementia* **4**(1), S137-S143 (2008). 10.1016/j.jalz.2007.10.008 18631990

[85] Guillem F, Rougier A, Claverie B (1999). Short-and long-delay intracranial ERP repetition effects dissociate memory systems in the human brain. J. Cogn. Neurosci..

[86] Schendan HE, Kutas M (2003). Time course of processes and representations supporting visual object identification and memory. J. Cogn. Neurosci..

[87] Hamberger MJ, Friedman D, Ritter W, Rosen J (1995). Event-related potential and Behavioral correlates of semantic processing in Alzheimer′ s patients and normal controls. Brain Language.

[88] Joyal M, Groleau C, Bouchard C, Wilson MA, Fecteau S (2020). Semantic processing in healthy aging and Alzheimer’s disease: A systematic review of the N400 differences. Brain Sci..

[89] Horvath A (2018). EEG and ERP biomarkers of Alzheimer's disease: A critical review. Front. Biosci..

[90] Bowling JT, Friston KJ, Hopfinger JB (2020). Top-down versus bottom-up attention differentially modulate frontal–parietal connectivity. Hum. Brain Mapp..

[91] Rugg MD, Yonelinas AP (2003). Human recognition memory: a cognitive neuroscience perspective. Trends Cogn. Sci..

[92] Preston AR, Eichenbaum H (2013). Interplay of hippocampus and prefrontal cortex in memory. Curr. Biol..

[93] Delbeuck X, Van der Linden M, Collette F (2003). Alzheimer'disease as a disconnection syndrome?. Neuropsychol. Rev..

[94] Li R, Nguyen T, Potter T, Zhang Y (2019). Dynamic cortical connectivity alterations associated with Alzheimer's disease: An EEG and fNIRS integration study. NeuroI. Clin..

[95] Berron D, van Westen D, Ossenkoppele R, Strandberg O, Hansson O (2020). Medial temporal lobe connectivity and its associations with cognition in early Alzheimer’s disease. Brain.

[96] Sun, P. *et al.* Mapping the cortical structural connectivity with super-voxel fiber connectivity density in Alzheimer's disease. 10.26044/ecr2020/C-05186 (2020)

[97] Guerra, A. *et al.* Transcranial Magnetic Stimulation Studies in Alzheimer's Disease. *Int. J. of Alzheimer’s Disease* **2011**, 263817. 10.4061/2011/263817 (2011). 10.4061/2011/263817 PMC3132518 21760985

[98] Walsh V, Cowey A (2000). Transcranial magnetic stimulation and cognitive neuroscience. Nat. Rev. Neurosci..

[99] Julkunen P (2008). Navigated TMS combined with EEG in mild cognitive impairment and Alzheimer's disease: A pilot study. J. Neurosci. Methods.

[100] Bergmann TO (2018). Brain state-dependent brain stimulation. Front. Psychol..

[101] Esposito R, Bortoletto M, Miniussi C (2020). Integrating TMS, EEG, and MRI as an approach for studying brain connectivity. Neurosci..

[102] Riley RD (2020). Calculating the sample size required for developing a clinical prediction model. BMJ.

[103] He (2019). Electrophysiological Brain Connectivity: Theory and Implementation. IEEE Trans. Biomed. Eng..

[104] Schoffelen JM, Gross J (2009). Source connectivity analysis with MEG and EEG. Hum. Brain Mapp..

[105] Zeng H-M, Han H-B, Zhang Q-F, Bai H (2021). Application of modern neuroimaging technology in the diagnosis and study of Alzheimer's disease. Neural Regener. Res..

[106] Trzepacz PT (2014). Comparison of neuroimaging modalities for the prediction of conversion from mild cognitive impairment to Alzheimer's dementia. Neurobiol. Aging.

[107] Ahmadzadeh M, Christie GJ, Cosco TD, Moreno S (2020). Neuroimaging and analytical methods for studying the pathways from mild cognitive impairment to Alzheimer’s disease: Protocol for a rapid systematic review. Syst. Rev..

[108] Zhang Y (2021). Alzheimer’s disease multiclass diagnosis via multimodal neuroimaging embedding feature selection and fusion. Inform. Fusion.

[109] Liu M, Cheng D, Wang K, Wang Y (2018). Multi-modality cascaded convolutional neural networks for Alzheimer’s disease diagnosis. Neuroinformatics.

[110] Lee Meeuw Kjoe PR, van AgelinkRentergem JA, Vermeulen IE, Schagen SB (2020). How to Correct for Computer Experience in Online Cognitive Testing?. Assessment.

[111] Bozionelos N (2001). Computer anxiety: Relationship with computer experience and prevalence. Comput. Hum. Behav..

[112] Di Giacomo D, Ranieri J, D’Amico M, Guerra F, Passafiume D (2019). psychological barriers to digital living in older adults: Computer anxiety as predictive mechanism for technophobia. Behav. Sci..

[113] Bong, W. K., *et al.* Designing nostalgic tangible user interface application for elderly people. In *Computers Helping People with Special Needs: 17th International Conference* Proceedings, Part II 17 471-479. (Springer International Publishing, Lecco, Italy, 2020).

[114] Lindberg, R. S., & De Troyer, O. Towards a reference model of guidelines for the elderly based on technology adoption factors. In *Proc. of the 6th EAI International Conference on Smart Objects and Technologies for Social Good* 30-35 (2020).

[115] Wilkinson C, Cornish K (2018). An overview of participatory design applied to physical and digital product interaction for older people. Multi. Technol. Interact..

[116] Borland D, Ii RMT (2007). Rainbow Color Map (Still) Considered Harmful. IEEE Comput. Graph. Appl..

[117] Rodin EA (1991). Some problems in the clinical use of topographic EEG analysis. Clin. Electroencephal..

