# Supplementary material for: Evaluating frontoparietal network topography for diagnostic markers of Alzheimer’s disease
Source: Sci Rep. 2024 Jun 19;14:14135. doi: 10.1038/s41598-024-64699-w (PMC11187222; doi:10.1038/s41598-024-64699-w)
Supplement: Supplementary file 1 — Supplementary Information 1. [file 41598_2024_64699_MOESM1_ESM.pdf]

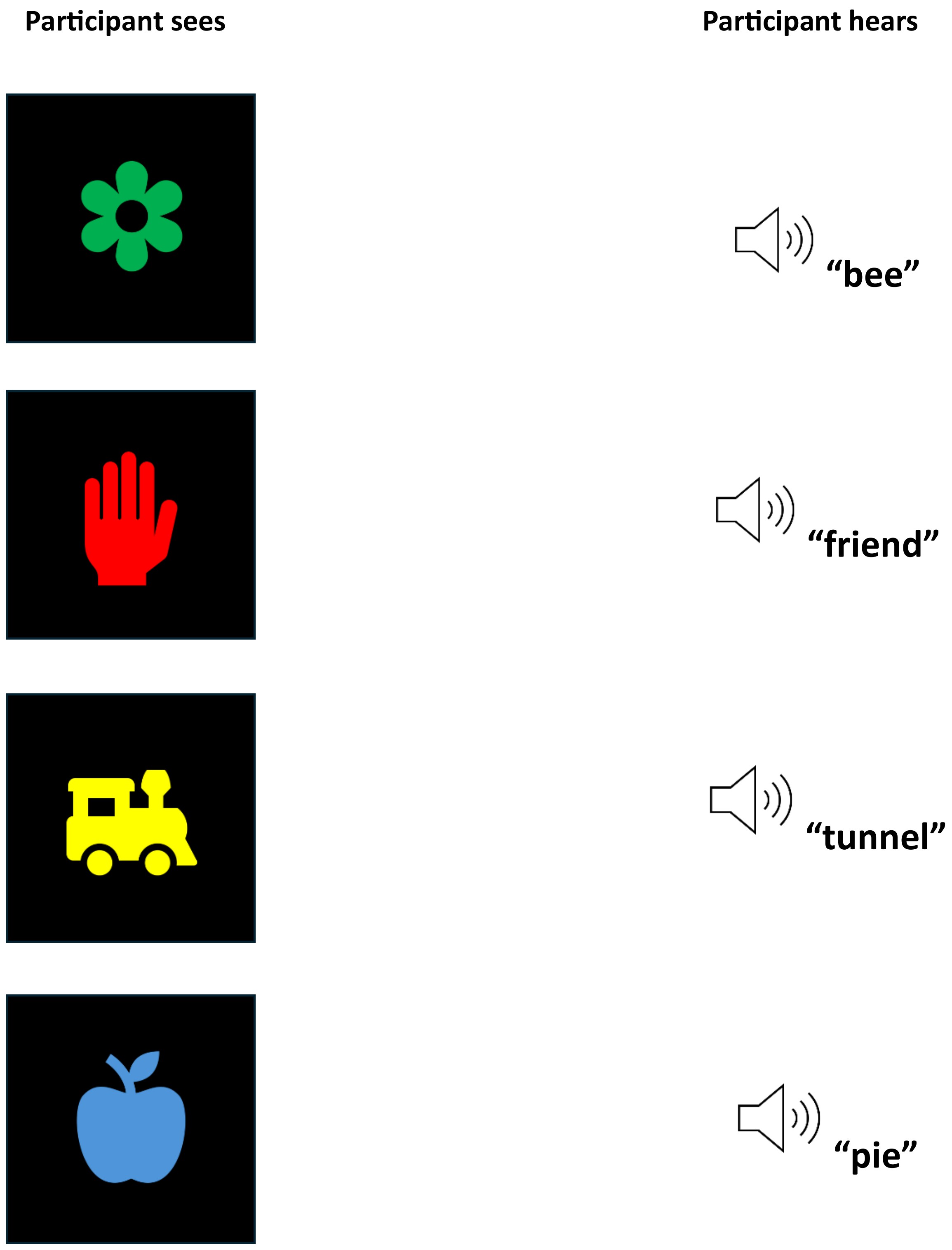

Figure 1. Alzheimer's Disease Evoked Potential Test (ADEPT). Associatively related stimulus pairs consisting of a synchronously presented picture and a spoken word presented on a computer screen. The ADEPT elicits hippocampal function in two ways: episodic memory and early integration of cross-modal sensory input.

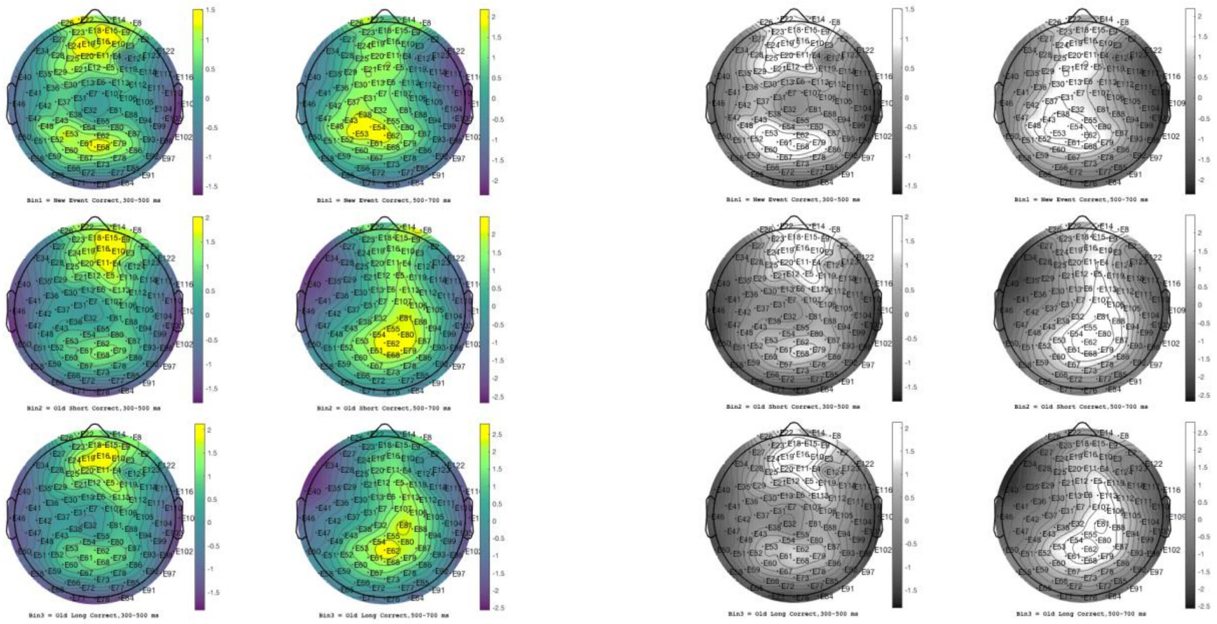

Figure 2. Grand average ERP scalp plots for (n=73) healthy controls, correct answers, average across 300-500 ms and 500-700 ms latency windows represented in lower contrast viridis and grey colormaps in EEGLab v2020.0. An example of a colour scheme argued by researchers to be more perceptually uniform.
